# Dr. Peaal, on Autumnal Fevers

**Published:** 1802-04

**Authors:** Tuaam Peaal

**Affiliations:** Aberdeen


### *To the Editors of the Medical and Physical Journal.*

GENTLEMEN,

IF you think the following obfervations on the Fever which raged in Aberdeen, deferving a place in your ufeful Publication, I fhall thank you to infert them.

I am, &c.

*Aberdeen, Feb.* 27, 1802. TUAAM PEAAL.

---

DURING the months of November, December, and January, fevers were very prevalent in Aberdeen; the weather in thefe months was very ftormy, and the froft intenfe, yet it had not the effect either of diminifhing their malignity, or ftopping their progrefs. Typhus and its varieties, efpecially that kind called Synochus, or a mixture of Synocha and Typhus, are, according according to Dr. Fergufon, the fpecies of Fevers which moft commonly affect the inhabitants of Aberdeen. He obferves, that every country has its own particular fpecies of Fever, which depends upon the nature of the place, and temperaments of the inhabitants; and this difference, he fays, appears in the feries and malignity of the fymptoms. The Fevers which commonly occur here, when they do not proceed from fpecific contagion, can be attributed to no other remote caufe than cold, which has by feveral authors been confidered as the moft frequent caufe. *This may account for Fever appearing more frequently in winter and cold weather, than when it is temperate and warm.*

The air round Aberdeen is in general pure and denfe, free from fogs and marfh miafmata; yet it frequently abounds with marine vapours, efpecially when the wind is eafterly, which is the caufe of fudden viciffitudes of heat and cold. This produces fthenic diathefis, and therefore frequently fubjects the inhabitants of this city to catarrh, rheumatifm, pneumonia, and other difeafes, the effects of a changeable atmofphere. Their food may alfo contribute to the fame effect, being denfe and coarfe; confifting principally of corn meal, which is very heating, and requires a ftrong and healthy ftate of the ftomach and chylopoietic organs to reduce it to chyle. The cool air of this place, along with exercife, is however very favourable for affifting the digeftion not only of the kind but alfo the quantity which is generally taken; and were they to be fed with food lighter, and more eafily digefted, they would complain of hunger in lefs than half an hour. Intermittents and remittents do never occur here. Dr. Fergufon, in his Effay on Fever, has alfo remarked this, we may therefore infer that not only the fevers which rage here, but their caufes, are fpecifically different from that of intermittent fever; and as a further proof of it, the air of this place generally proves a cure for the latter. Dr. Brown fays, that in Typhus there is great accumulation of excitability, want of excitement, and deficience of the vital powers or defective ftimuli. Dr. Cullen, that there is a great degree of fpafm, and violent re-action. Dr. Fergufon thinks the debility is owing to the incapacity of the fyftem for receiving and retaining oxygen, which he fays is diminifhed in Typhus. But we muft confefs that, notwithftanding the light which thefe authors have thrown upon the fubject, we yet remain in darknefs. It is true, we are poffeffed of a medicine that will generally ftop the paroxyfm of intermittents, but we are not yet in poffeffion of any medicine that will arreft the progrefs of the continued paroxyfm, Has experience yet furnifhed us with a proper method of cure that will anfwer in every cafe of the fame kind of fever? Digitalis, mild muriated mercury, acids, and oxygen have been much recommended; yet, when tried, the advantage has not been greater than that attending the former methods of treatment. Neither is this to be wondered at, confidering that all the functions are fo violently affected in continued fever, that we are at a lofs to form proper indications of cure. The manner in which the caufes operate are hid from our view, and what is taken for caufes are only effects of the caufe. On this fubject a wide and extenfive field for improvement lies open. Nothing tends more to forward our knowledge of fever than having an exact account of the nature, caufes, and cure of fuch fevers as are endemic in particular places. By this we have already learned that contagion and cold temperature, operating upon the inhabitants of certain places, produce fevers of the typhoid kind; and that in warm climates, contagion and heat operating will produce a fever of a different and more infectious nature, like the bilious remittent fever. It is known too, that marfh miafmata, acting with cold, will produce a certain fpecies of intermittent; when acting with heat it will produce a different kind; hence the well-known diftinction of intermittents into vernal and autumnal. Dr. Fergufon, with great juftice, confiders that cold and heat frequently act as remote caufes, and that excefs of heat or cold obftructs the proper oxydation of the fyftem. Whether or not this be the cafe, we find cold acting in excefs to have been the moft frequent caufe of the fever which raged in Aberdeen during the paft winter. It moft frequently affected the young and growing; it commenced with languor and chillnefs, flight pain in the knees, fmall of the back, and forehead. The fenfation of cold was fometimes very violent, but feldom produced tremor. From the beginning there was great diminution of ftrength, attended with anxiety and dejection of fpirits. From the weaknefs of the mufcular action of the thorax, the patient complained of oppreffion at the breaft, accompanied with frequent fighing, and an averfion to fpeak. They complained of a fenfation in their ftomach as if they had eaten an over meal; this was attended with more or lefs ficknefs, naufea, and fometimes vomiting. Thirft, drynefs of the fkin, fcanty and high coloured urine likewife attended. The chillnefs generally continued two or three days, and was fucceeded by a warmnefs of the fkin, which increafed to a burning heat over the whole body, fenfibly felt by every perfon who touched the patient; and if any perfon flept with him he felt a difagreeable fenfation of a dry intenfe heat, as if the patient's fkin were burning him. This heat was very fevere, and continued for a longer or fhorter period according to circumftances. It often continued four or five days days without any fign of fweat appearing, and during this ftate of heat the patient felt himfelf as uneafy as in the ftate of cold. There was alfo a flight rednefs of the adnata of the eyes, attended with throbbing of the temples, furred tongue, quick pulfe, and refpiration. Sometimes a kind of moifture arofe upon the fkin, but it more frequently was dry and parched. This fweat feldom gave any relief to the fymptoms, and when it was profufe it generally tended to weaken the patient more. Delirium, in fome cafes, came on about the fixth or feventh day, in others not till the tenth. This fymptom was very variable, fometimes it was violent, and at other times it occurred only during fleep; it was accompanied very frequently with fubfultus tendinum, difficulty of uttering words, and comatofe ftupor. Thefe fymptoms, however, only attended the worft cafes. When the delirium was violent, if it did not abate within three days, the patient feldom recovered. The crifis happened about the 15th, 16th, or 17th day. When favourable, the pulfe became foft, regular, and not fo quick; the exceffive heat and thirft abated, and a moifture appeared on the fkin; the delirium and fubfultus tendinum alfo abated, and the patient was more inclined to fleep. When unfavourable, the delirium and comatofe ftupor increafed, the pulfe turned very quick and feeble, the patient toffed much and fometimes cried, would not take drink, became incapable of fpeaking, and at laft death clofed the fcene. This is the manner in which the fever began, proceeded in its courfe, and terminated either in health or death. Sometimes the fymptoms would vary a little, but in general the deviation was very inconfiderable. A diarrhœa would in fome cafes appear and prove troublefome, fometimes alfo flight hemorrhage from the nofe; but thefe fymptoms did not always attend, nor did they ever prove favourable.

The moft fuccefsful method of treating this fever was the following: When the patient applied for affiftance in the beginning of the fever, or during the time when he felt his body chilly and cold, he was ordered to bed, and a cup full of warm water, a little fweetened, given him every hour, in which was mixed ten gts. of a mixture of equal parts of vinum antimonii and tinctura opii, until he began to perfpire; at the fame time a blifter was applied to each temple, which always had the effect of removing the pain of the head. After twelve hours, if this treatment had not produced a gentle perfpiration, but the burning heat ftill continuing upon the furface of the fkin, the patient was bathed with cold water, his body well dried, and an anodyne draught prefcribed. The drink was now changed to weak negus, with the addition of half the quantity

 formerly formerly prefcribed of the vin. antimon. & tinct. opii. If thefe means were attended with the effect of keeping up a gentle perfpiration, nothing elfe was given excepting chicken or veal foups which were ufed as food. When, however, the fever, burning heat, ficknefs, quick pulfe, ftupor, and delirium increafed, the quantity of wine was augmented, and ftimulant cataplafms applied to the feet, fuch as recommended formulæ 241 by Dr. Fergufon, with the following bolus from the fame author repeated every four hours until the fever begins to abate. ℞. G. camph. fuccinat potafiæ aa gr. iij elect. theb. gr. v. ft. bolus.—When cough proved troublefome the fevum ceti mixture was had recourfe to. When diarrhœa was obftinate, attended with a dry and hot fkin, affufion with cold water, giving at the fame time fmall dofes frequently repeated of the tinct. opii, was found very beneficial.

